# Simulated Evolution of Signal Transduction Networks

**DOI:** 10.1371/journal.pone.0050905

**Published:** 2012-12-12

**Authors:** Mohammad Mobashir, Burkhart Schraven, Tilo Beyer

**Affiliations:** 1 Institute of Molecular and Clinical Immunology, Otto-von-Guericke University, Magdeburg, Germany; 2 Department Immune Control, Helmholtz Centre for Infectious Disease (HZI), Braunschweig, Germany; Semmelweis University, Hungary

## Abstract

Signal transduction is the process of routing information inside cells when receiving stimuli from their environment that modulate the behavior and function. In such biological processes, the receptors, after receiving the corresponding signals, activate a number of biomolecules which eventually transduce the signal to the nucleus. The main objective of our work is to develop a theoretical approach which will help to better understand the behavior of signal transduction networks due to changes in kinetic parameters and network topology. By using an evolutionary algorithm, we designed a mathematical model which performs basic signaling tasks similar to the signaling process of living cells. We use a simple dynamical model of signaling networks of interacting proteins and their complexes. We study the evolution of signaling networks described by mass-action kinetics. The fitness of the networks is determined by the number of signals detected out of a series of signals with varying strength. The mutations include changes in the reaction rate and network topology. We found that stronger interactions and addition of new nodes lead to improved evolved responses. The strength of the signal does not play any role in determining the response type. This model will help to understand the dynamic behavior of the proteins involved in signaling pathways. It will also help to understand the robustness of the kinetics of the output response upon changes in the rate of reactions and the topology of the network.

## Introduction

Signal transduction is a critical step in inter- and intra-cellular communication [1]. In signal transduction processes, an external stimulus is transformed into a cellular response through a network of proteins that ultimately alters the function and behavior of the cell [2], [3]. Different forms of input-output relationships shown by biological signal transduction are known from experimental work [4], [5]. The change of input signal strength, kinetic parameters, or the network topology can give rise to sustained, oscillatory, or adapted responses. These different types of response underlie the specialized functions of cells such as proliferation, differentiation, and apoptosis [6].

An example for a pathway that shows different response types depending on the cell type and/or stimulus is the mitogen-activated protein kinase (MAPK) pathway. This pathway involves Raf, MEK (MAPK/ERK kinase), and ERK (extracellular signal-regulated kinase) and is considered to be centrally involved in cellular decision making processes where small quantitative differences often lead to major phenotypic changes [7], [8]. It has been shown that the upstream molecules induce quantitative and qualitative differences in the duration and magnitude of ERK activity that regulate the function and behavior of a cell [6]. The MAPK pathway is a prototype for the general scheme of signal transduction, in which after receiving a signal from ligand-bound receptors, the involved proteins are altered (“activated”) by post-translational modifications [9]–[11]. Subsequently, the active form activates other inactive proteins by means such as recruitment to specific locations, altering the enzymatic activity, or conformational changes exposing binding sites for further binding partners. To predict the function of a signaling module it is necessary to understand the design principles of signaling networks (SNs) that underlie the behavior, function, and robustness [12]. From experiments neither the topology of a SN nor the kinetic parameters of its underlying elementary interactions are known in detail such that it remains open how sensitive the function of a network is to these parameters. Therefore, it seems appealing to explore the evolution of signaling networks allowing mutations of kinetic parameters, changes in the network topology such as the addition of new proteins to mimic the subsequent acquisition of additional regulatory layers.

In previous studies, various modeling approaches have already been applied to investigate the behavior of SNs. Francois and Hakim (2003) [13] evolved genetic circuits to produce a variety of functional behaviors and demonstrated the vital role of post-transcriptional interactions, i.e. protein-protein interactions controlling gene regulation. This evolutionary approach has been extended by others to protein-protein interaction networks with specific functional characteristics: oscillators, bistable switches, homeostatic systems, and frequency filters [14], [15]. Yet, none of these approaches investigated in how far the formation of transient protein-protein complexes influences the generated networks or whether association, dissociation, or catalytic rates are critical for the network properties.

In this study, we used ordinary differential equations (ODEs) to describe the dynamics of nodes which represent the proteins and transient complexes forming a SN. The focus of this study is mainly on the evolution of a SN's response due to variations in the kinetic parameters or addition of new nodes when faced with the basic task of detecting the presence of a signal by generating an above-threshold response with arbitrary kinetics. We find that the detailed parameter values are not critical for the functional response of the network. The interaction strength influences the sensitivity of the network, i.e. whether to respond or not to an input of given strength, rather than whether to respond with a transient or sustained output.

## Results

We start the evolution of SNs by assuming that the basic task of signal transduction is to provide an above-threshold response to a signal which is generated by a ligand binding to its receptor (see Methods). The response is measured at a pre-selected node. When the activation state of this node crosses a threshold the network for an arbitrary time, the SN is considered to be successful in the detection of the signal and the fitness is increased by 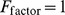 otherwise the fitness for the signal in question remains 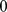. For the task to detect multiple signals of different strengths the fitness contributions are summed up. In our simulations signals are either present throughout the simulation or given as a pulse of fixed duration.

**Figure 1:**
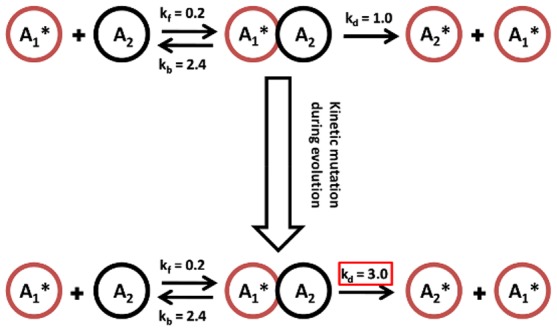
Mutation of kinetics parameters. 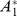
 represents the active form of the protein 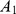, 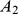 another inactive protein molecule, 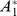 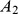 is the complex formed during the reaction between 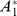 and 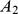. 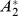 is the active form of 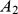. 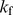, 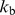, and 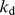 are the rates (interaction strength) of the reactions. A mutation of the reaction alters any of the rates, e.g., 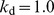 (top) adopts the new value 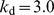 (bottom).

**Figure 2:**
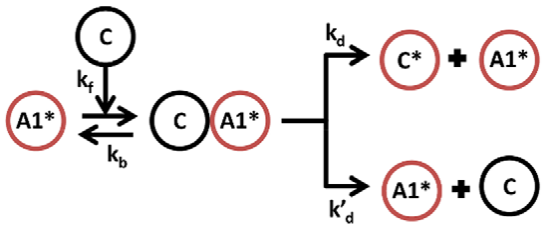
Addition of a new node in the minimal model. An inactive protein C is added, that can either become activated (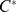) by 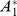 (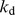) or is inactivating the active protein 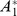 (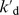).

We investigate the evolution of SNs and their dynamic activation pattern using two types of mutations: Variations of kinetic parameters (Fig. 1) or addition of new nodes (Fig. 2 and S3). The evolution with both mutation types are investigated independently and compared using replicates with identical parameter settings but different seeds for the pseudo-random number generator.

### 3.1 Evolution of SNs with varying interaction strength

**Figure 3:**
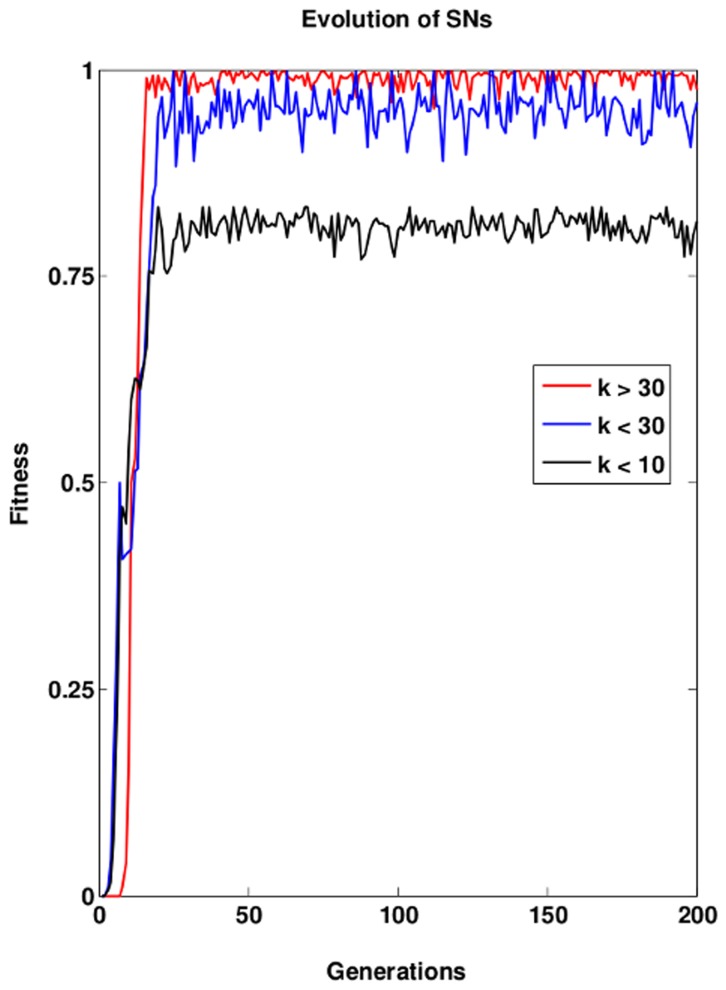
Evolution of SNs by mutating kinetic parameters. The fitness (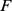
norm) during the evolution of SNs for three different regimes of maximal interaction strengths: weak (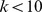), moderate (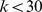), and strong (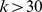) interactions (Number of generations: 200, Number of SNs: 200, Threshold level: 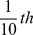 of the initial concentration level of protein).

We analyze the effect of strong and weak interactions by simulating SNs evolution for three different regimes: weak (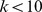, dimensionless parameter), moderate (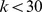), and strong interactions (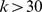). When the interaction strength remains below a certain value (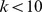 in our model setting) the networks are unable to reach maximum fitness (Fig. 3). At the same time the fitness of the population fluctuates significantly. If the interaction strength remains below a value of 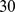, then the networks population reaches almost maximum fitness, but exhibits a considerable amount of fitness fluctuations. Further increase in the interaction strength (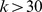) suppresses fluctuations in the fitness, i.e., a population has evolved in which virtually all the networks are able to detect every single input signal. For all parameter regimes we observe that the evolutionary process approaches a steady state after less than 30 generations (Fig. 3).

### 3.2 Strong interactions promote signal strength-independent and robust activation patterns

**Figure 4:**
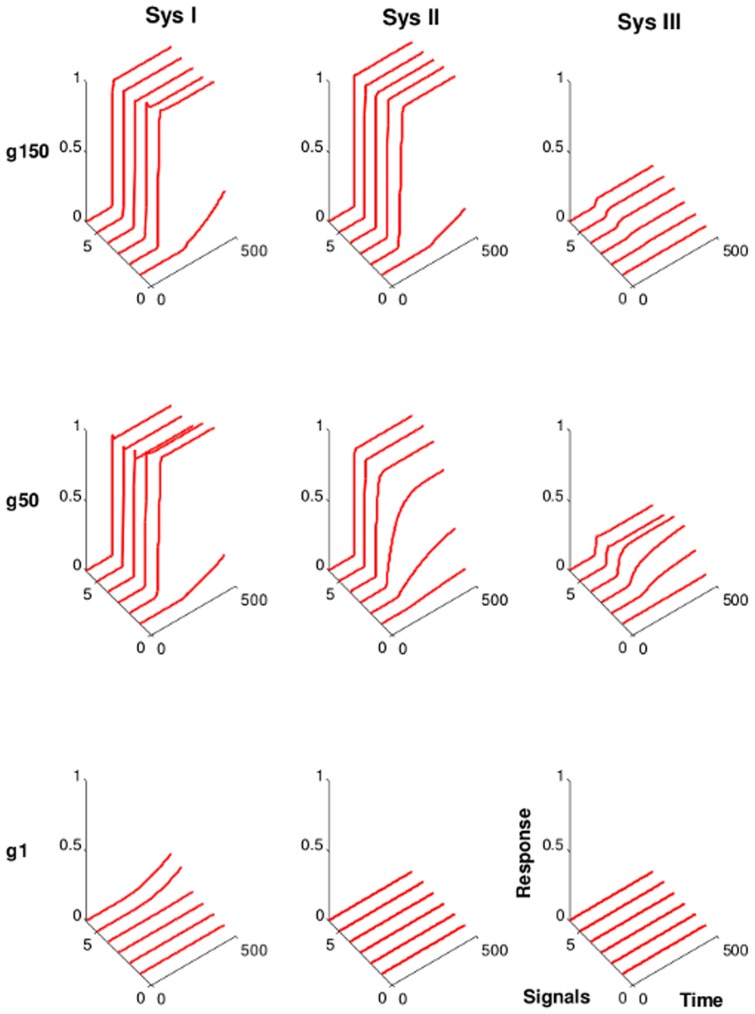
Kinetics of the evolved networks. Shown are the networks with the highest fitness score from one of the simulation runs. 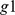, 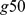, and 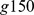 denote the generation 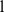, 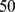, and 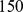, respectively. The six solid lines show the kinetics of activation of the output node in response to six different input signal strengths (strength increases with signal index). Signals are provided at time 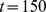. Sys I, weak interactions (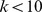). Sys II, moderate interactions (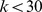). Sys III, strong interactions (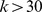).

In order to understand how the evolved networks manage the task of detecting signals, it is important to analyze the dynamic behavior of the networks over the evolution period. Due to the random generation of the initial kinetic parameters, the activation patterns of the nodes of the networks are different in each starting population. In analogy to our fitness function, we define a protein to be strongly active when its relative fraction in the active state passes a given threshold. Any other non-zero value defines the node as weakly active. We observe that the output node initially passes the signaling threshold only for the stronger signals while during the course of evolution the networks detect more and more signals (Fig. 4). Depending on the strength of interactions between the proteins and their complexes, most successfully evolved networks show a similar activation pattern with little change during the following generations. When the networks evolve and kinetic parameters are allowed to mutate within the range of 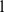 to 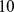 then the activation pattern is weak even with respect to strong input signals. Also the initial variable activation pattern remains throughout the entire evolution period (Sys I in Fig. 4). Hence, weak interactions do not produce signaling strength-independent activation patterns. From this observation we conclude that the kinetics of the output nodes of the evolved networks are not robust when proteins interact weakly. The same topology, however, can detect signals when the interaction strength is increased. Evolution of networks with kinetic parameter values in the range of 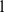 to 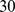 show strong activation and also display a similar activation pattern throughout the SN population after about 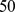 generations. If networks are evolved permitting even higher kinetic parameter values (k>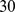) then the networks quickly adapt their dynamics to strong and robust activation patterns. The output node of each of the successful network becomes activated soon after detecting the signal and shows almost equal response strength and similar activation pattern irrespective of the input signal strength (Sys II and III in Figure 4).

**Figure 5:**
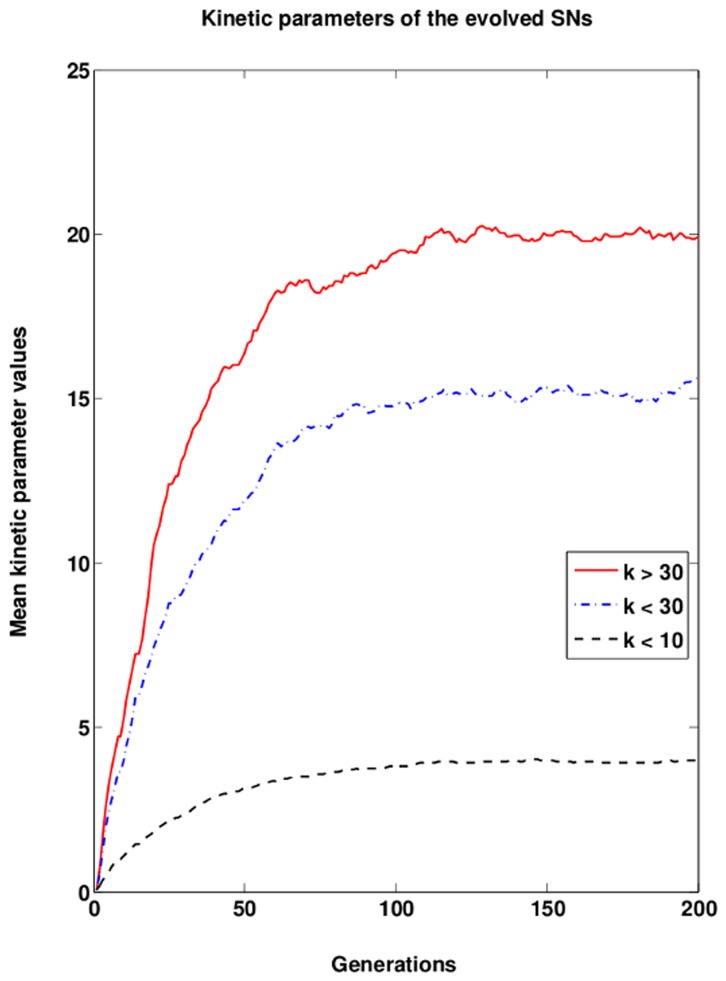
Mean kinetic parameter values of networks during evolution. The numeric values for all kinetic parameters in the SN are averaged omitting the formal difference of first-order and second-order reactions (see Methods).

In our simulations, we observe that about 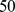 generations are sufficient to achieve a stationary distribution of activation patterns all of which show strong activation provided the interaction strength is sufficiently high (Fig. 4). Yet, an investigation of the mean kinetic parameter shows that we have a drift towards stronger interactions in the following generations (Fig. 5). It takes as long as 150 generations to achieve a stationary population for the regime in which the strongest interactions are permitted (Fig. 5). A detailed investigation of the parameter distribution of the final generation shows that there is no preferred pattern of kinetic parameters (Fig. S1 and S2). In a brief phase around the time when the population reaches maximal fitness the population is dominated by the networks having rather similar kinetic parameters (Fig. S2). Subsequent generations then diversify again resulting in a wide distribution of kinetic parameters used by the networks (Fig. S2). This suggests that none of the interactions is critical in the sense that it is subject to a strong selection pressure. Moreover, the same topology can solve the task using very different parameter setups which appear to form a connected set in the parameter space given the chosen fitness function. However, a further increase in the interaction strength does not provide a selective advantage as it has only minimal influence on the activation pattern of the network.

### 3.3 Dose-response relationship of the evolved SNs

**Figure 6:**
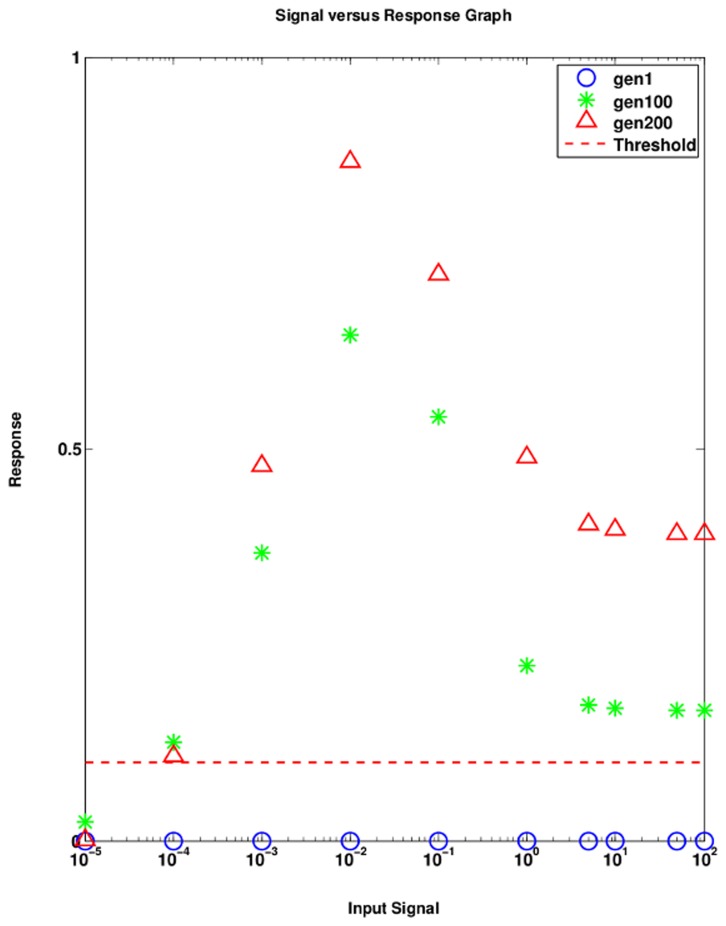
Dose-response relationship of the evolved SNs for the strong interaction regime. The graph shows the input signal versus the maximum response of the output node of the best network in the respective generations 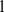, 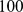, and 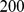.

The activation patterns evolving when strong interactions are permitted appear independent of the input signal strength, a behavior also known for the MAPK cascade in certain systems [16]. We extended our analysis to investigate the dose-response of the evolved networks beyond the range of signaling strength used for selection. Before evolution, at very weak input signal, the networks do not produce a considerable response with respect to the threshold (Fig. 6). After a few generations, all the networks are able to evolve and show strong activation even at very low input signal strengths (Fig. 6). Yet, the networks require a signal to become active confirming that the evolution did not generate self-activating networks, which would not be excluded by our choice of the fitness function. Thus, the strength of the input signal does not affect the activation pattern of the SN. Therefore, it appears that the generic behavior of a SN is switch-like when facing the task to ‘somehow’ detect a signal.

### 3.4 Effect of removal of input signals on the kinetics of the evolved SNs

**Figure 7:**
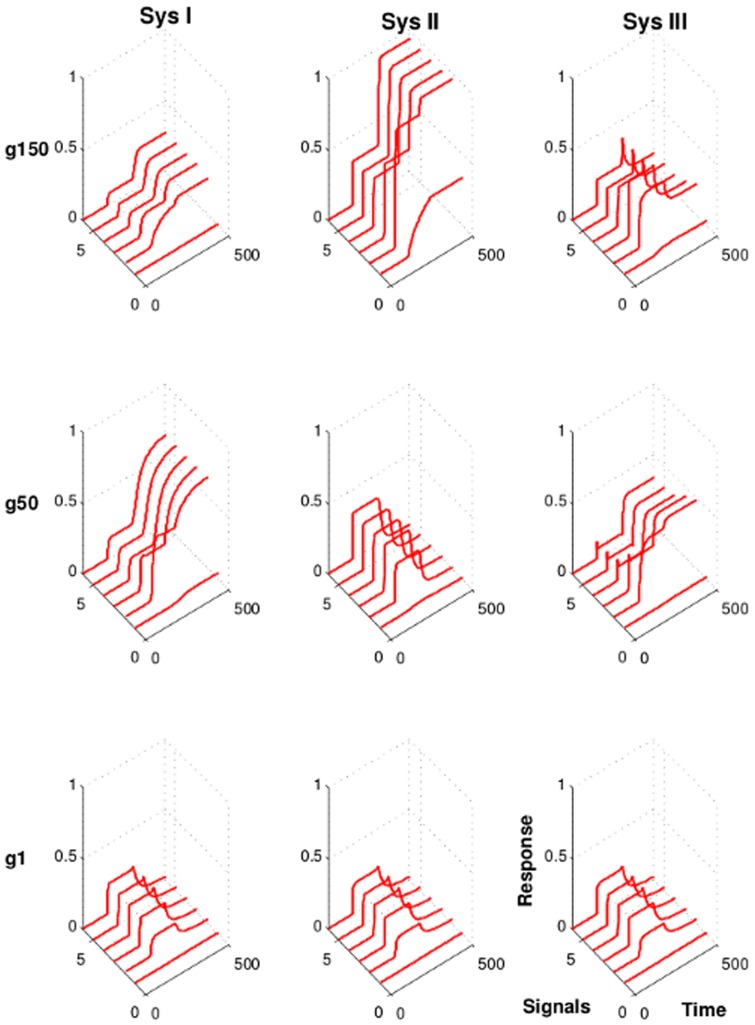
Effect of the removal of the input signal on the kinetics of evolved networks. Shown are the networks with the highest fitness score from one representative simulation run. 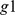, 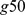, and 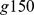 denote the generation 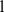, 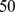, and 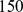, respectively. The six solid lines show the kinetics of activation of the output node in response to six different input signal strengths (strength increases with signal index). Sys I, weak interactions (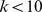). Sys II, moderate interactions (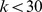). Sys III, strong interactions (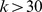).

To further investigate the behavior of the output node, we simulated pulse activation of the networks: After initial equilibration of the network the signal is present for a fixed amount of time before being removed again. Before the pulse is given, all the networks remain in their basal inactive state. Evolved networks typically respond with a switch-like response to the signal pulse (Fig. 7). Some of the networks will show an even enhanced response after the signal has been removed (e.g. Fig. 7), while the majority revert back to the initial state. Since, our fitness function does not generate selection pressure to either of the network responses after removing the signal, both response types are valid. It is interesting to observe the occurrence of a pulse-detector, which requires a memory of previous signals, e.g. by generating irreversibility in the system. With a fitness-function sensitive to the phase following the removal of the signal, a trigger or an irreversible switch could be easily selected from the networks generated in our simulations.

### 3.5 The role of partially active nodes

**Figure 8:**
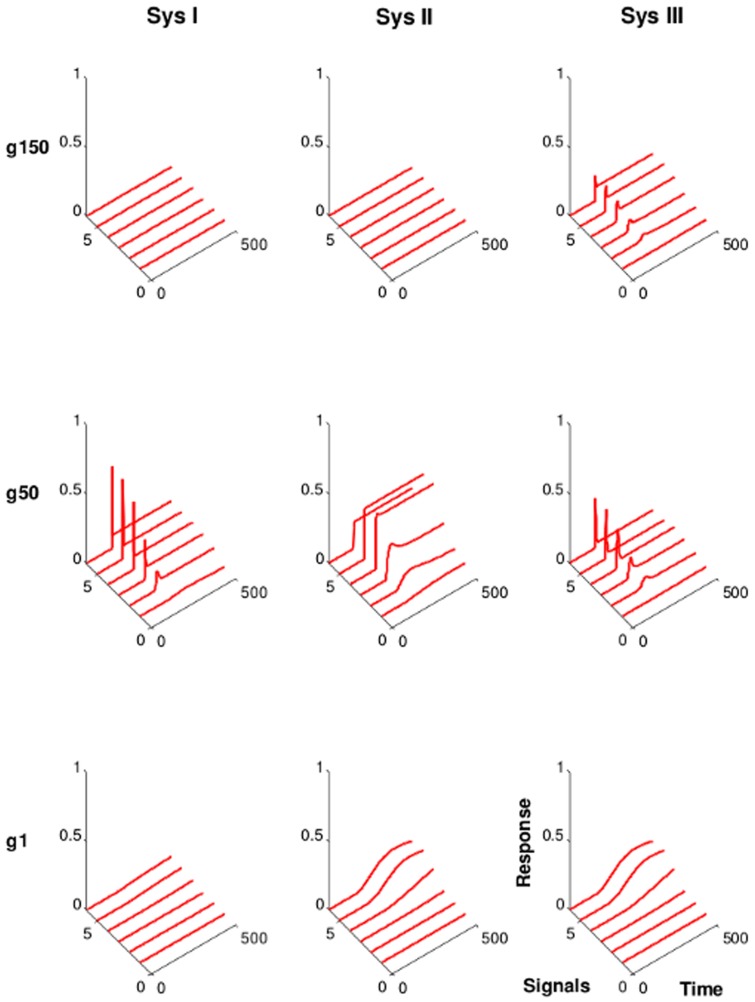
Role of the partially active nodes. Shown is the kinetics of the node (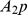) from the networks with the highest fitness score from one of the simulation runs. 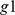, 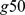, and 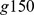 denote the generation 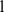, 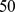, and 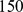, respectively. The six solid lines show the kinetics of activation of the output node in response to six different input signal strengths (strength increases with signal index). Sys I, weak interactions (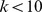). Sys II, moderate interactions (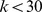). Sys III, strong interactions (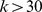).

After studying the kinetics of the pre-defined output node (a fully active node) we also studied the kinetics of the partially active proteins. We observed that single phosphorylated proteins show predominantly a transient response and some of the networks shows partially adapted response (Fig. 8). There is a clear trend that weaker interaction permit stronger transient activation of the monophosphorylated forms (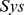 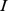, 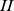, & 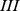 in Fig. 8).

### 3.6 Evolution of SNs by adding new proteins

We next evolve our randomly generated SNs by adding new proteins instead of altering kinetic parameters. The new proteins are randomly interacting with potentially all proteins in all states (but not complexes) with also randomly generated kinetic parameters (Fig. 2 and S3). Hence, all kinetic parameters are fixed as soon as the proteins are added. All other parts of the evolutionary algorithm remain the same.

The addition of new nodes displays virtually the same effects on the evolution of the networks as well as the dynamics of the response which we observed due to the mutation of kinetic parameters. After a few generations, all evolving networks show similar and strong activation patterns provided the new interactions arising with the newly added proteins are of sufficient strength (data not shown). Also the distribution of the kinetic parameters of the new nodes shows no trends towards a particular pattern.

## Discussion

**Figure 9:**
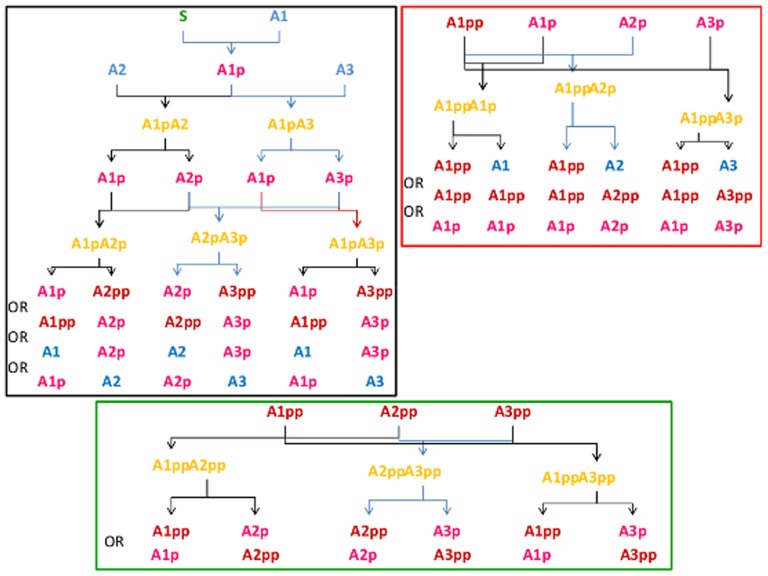
List of possible reactions in the minimal model with three proteins. 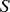
 represents the input signal, 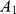, 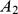, and 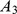 denote inactive signaling proteins, their partially active (single phophorylated) forms are 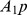, 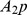, and 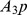, respectively. The fully active forms are 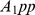, 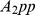, and 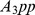.

We investigated the evolution of SNs under the premise that the primary task of signal transduction is to detect a signal without pre-determining a desired kinetics. As shown in Figure 9, any form of the protein can - depending on its interaction partner - play the role of a kinase or phosphatase. Typically, proteins do not fulfill both functions, however, due to their phosphorylation state may recruit proteins that perform this function but are not explicitly modeled in our approach. We simulated the evolutionary process by allowing mutations either in the kinetic parameters or the topology of the network. The SN population achieves maximum fitness only when protein-protein interactions are sufficiently strong. The generic solution is a sustained activity of the output node as long as the signal is present. Weak interaction strength results in networks that respond differently and only to some of the signals. In a cellular system weak interactions could therefore probably not provide reliable cellular decisions. For the input sensor - response relationship, we conclude that neither the starting topology nor the set of kinetic parameter values is constraining the evolution of the networks (provided sufficiently strong interactions). Therefore, short circuits coupling the receptor directly to the output node by two reactions are possible but certainly not the only solutions. In particular, the final generation has a high variability in the kinetic parameters suggesting that no dominant subnetwork of interactions exists.

According to previous published work, we can say that there are four different possible input-output relations for signal transduction [17]. The first one is the classical case which is single input and single output, the second possible relation is signal concatenation (multiple inputs and single output), the third relation is signal pleiotropy (single input and multiple outputs), and last one is the complex signaling event which has multiple inputs and multiple outputs. Out of all these four possible signaling events our model is designed to represent the classical case which is frequent in biological signaling processes. Although not the scope of our present study our model can also be modified to understand the remaining three types of signaling input-output relations in the future.

The activation pattern of the evolved networks in a stationary population are robust against strong interactions and in most cases sustained response, suggesting that this type of response is the generic cellular behavior when the presence of a signal is sufficient information for a cell [18]. We observe that the response kinetics does not alter after about 30–50 generations but the kinetic parameters still change. We interpret this in the following way: When the first networks with enhanced fitness appear they give rise to multiple clones that have largely similar kinetic parameters. This is similar to the bottleneck effect, i.e. many networks do not generate offsprings due to their low fitness and only similar networks pass on to the next generation. Following this phase the networks start to diversify again and a large range of the allowed kinetic parameter regime is explored. The diversification also indicates that there is a large number of solutions in the parameter space to ‘solve’ the fitness function. These solutions appear to be connected in a large set as the different kinetic parameters can be explored by the SNs without losing their fitness. Alternatively, one can view this situation as overfitting as the quite large number of parameters allows the networks to ‘solve’ the fitness function in many different ways.

As the sustained response appears to be the generic cellular response, we speculate that more complex responses such as analog dose-responses, adaptation, multi-stationarity, and oscillations require additional selective pressure. In particular, for analog responses that appears surprising, as one may expect that stronger signals lead also to stronger responses as often observed in experimental systems. Previous studies [13] demonstrated that the variation of kinetic parameters and addition of nodes is sufficient to evolve the networks that have a defined output response. However, this required corresponding fitness functions that encode the mathematical property of the desired system, e.g., bistability. These models can only be applied to those systems which are known to have such behavior, but often the exact behavior of the SNs is unknown. Therefore, the creation of a fitness function that encodes the task that a cell solves under certain experimental conditions, may be more beneficial in determining possible and likely behavior of the underlying SNs.

The higher average fitness of networks with strong interactions is due to the ability to detect weak signals. This corresponds to situations such as bacterial chemotaxis and T-cell receptor signaling where cells are sensitive to detect very few ligands. In the simulated systems, we have three different simulating conditions. weak (k<10), moderate (k<30), and strong (k>30). Signaling networks are still functional with or moderate interactions. However, at weak interaction strengths the SNs will work but in case the input signal is also weak then SNs often fail to detect. When the interaction strength is moderate then the SNs function and can also respond to weak input signals. Another difference between the SNs working at moderate and strong interaction strength is that at strong interaction strength the SNs show a strong and quick output response in contrast to weak interactions where the kinetics is typically slow and weak.

The rapid increase in fitness for the SNs suggests that any weakly interacting network that is capable of evoking at least some response to a signal, quickly evolves into a strongly interacting SN provided the selective pressure is present. This results in a high flexibility of cells to gain new signal transduction pathways when required and the critical invention is the proper receptor rather than a correct connection to the appropriate cellular response. Thus, a cell may retain a number of weak interactions among signaling proteins that do not interfere with primary signaling pathways, which can be converted should such a demand arise during evolution. As a consequence, a high number of weak unspecific interactions among proteins enables the cell to flexibly and quickly adapt to changing environments. Based on our results, we hypothesize that this is not a property that must be developed by a cell during evolution, but is inherent to weakly interacting protein-protein interaction networks. The diversification of kinetic parameters following the evolution of successful SNs in the regime of strong interactions also indicates that a large number of weak interactions do not harm the performance of the evolved signal-response relation. Thus, the SNs can reliably respond to the signal while at the same time retain a plethora of connections which may be used to ‘solve’ evolutionary demands that may occur in addition. This effect is in agreement with the notion that robustness combined with a high evolvability is a favorable and likely outcome of evolution [19]–[21].

At present, the model evolves the interaction strength and topology of the SNs. The kinetic parameters are in general hard to access while the topology is comparatively easy to analyze experimentally. In our simulations, we see that the exact values of the kinetic parameters play a minor role. Unfortunately, the number of possible parameter sets to generate a certain behavior is large such that the topology alone is not likely to predict the function of the network reliably. However, it is not only the interaction strengths and input signals which vary but also protein concentration levels affect the behavior of a SN [7], [22] and in addition bear the advantage of being experimentally quite easily accessible variables. Furthermore, stimulation of receptors virtually never occurs in isolation and therefore the interaction of signal transduction pathways can become relevant [17], [23], [24]. The questions of how does cross-talk affect the network's behavior and how does it affect the evolution of the SN is worth pursuing using our approach by embedding a SN into the wider context of co-evolving signaling networks.

In the future, it will also be interesting to investigate the role of random fluctuations, at the receptor level during ligand receptor binding (external noise) and stochastic fluctuations in the signaling network (internal noise). with three different types of noise: (i) random fluctuations at the receptor level in the absence of a signal (external noise) (ii) variations of the signal (i.e. signal+noise), and (iii) stochastic fluctuations in the signaling network (internal noise). For case (i) we already show in this study that a certain amount of signal is required to create a response in the network, i.e. any noise below the threshold will not induce a response as defined by passing a threshold activation level of the output node. The fact that the output kinetics is virtually independent of the input signal indicates that noise added to a sufficiently large input signal (case (ii)) will not be detected by the cell corresponding to a situation previously analyzed [25]. In line with this, any change of the output response that happens above the threshold cannot account for biological effects as the fitness functions does only resolve above- and below-threshold responses. If cells need to do so, one would need a refined fitness function reflecting the additional features. Therefore, an investigation of the evolution of the networks using a stochastic approach will be required in order to analyze the impact of internal noise (case iii) on the evolution of SNs.

## Conclusion

From our results, we conclude that sustained responses are the generic solution of a SN when the mere detection of the presence of a signal is relevant. This response occurs as soon as the protein-protein interactions are of sufficient strength, either by mutation of the kinetic parameter underlying existing interactions with the network or by recruiting new proteins to the network that generate a sort of bypass by supplying the network with strong interactions. Remarkably, the exact values of the kinetic parameters are irrelevant as soon as a pathway of sufficiently strong interacting proteins is provided. Given the quick evolution of the SNs, we conclude that weak protein-protein interactions serve as a pool to rapidly evolve new pathways, but play only a minor role in modulating the actual responses of a signal transduction network.

## Materials and Methods

### 6.1 Model

We set up a simplified model to represent a signal transduction pathway allowing two post-translational modifications of similar to the MAPK cascade [26]. In order to transduce the signal, we have included protein-protein interactions, protein phosphorylation and dephosphorylation [27]. Double phosphorylated proteins act as fully activated and single phosphorylated molecules as partially activated molecules. Note, that the term phosphorylation is used for convenience as any other post-translation modification adding a small chemical group, lipid, protein or carbohydrate modifying a protein's spectrum of interaction partners or enzymatic activity are covered by the model.

The interaction between the signaling proteins are set up randomly to create the initial population as well as when adding proteins during evolution. In our current model, we have not classified the proteins of the SN. The kinase or phosphatase function of a protein is determined for each reaction by the matrix 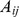 (see below). Initially, all the proteins are inactive. One of the initially present inactive signaling molecules is designated as receptor and receives the signal to become activated. The total number of input signals are six and each network is tested for their response to these six different input signal strengths. Once the receptor receives the signal then it can activate other signaling molecules. All the signaling molecules are allowed to phosphorylate or dephosphorylate each other (Fig. 9) and the final products will be formed depending on the complex. All the reactions in this model are bimolecular, autophosphorylation and homodimer formation are not allowed. Every molecule that becomes partially (single phosphorylated) or full active (double phosphorylated) can interact with any other molecule in any state. These interactions lead to complex formation. The complexes can dissociate without changes to its constituents or upon modifying on of it by means of phosphorylation or dephosphorylation. The interaction of two partially active molecules produces either one of them being fully activated (dual phosphorylated) or inactivated (dephosphorylated) without changing the other reacting partner's state (attributing it an enzymatic role) as shown in Figure 9. Which of the possible reactions are realized is determined randomly once at the beginning (with constraints, see next paragraph), thus setting up the network topology. In addition, one randomly selected double phosphorylated protein, different from the receptor node, is designated the output node. It represents the molecule which will eventually induce the cellular response.

### 6.2 Generalized mass action kinetics equation

A network consists of the above mentioned signal transduction pathway where both inactive and active proteins and complexes are represented as nodes. The interaction matrix (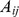) between all these molecules including complexes are represented as 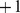 (production/generation), 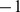 (degradation/dissociation), and 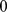 (no interaction). The entries of 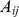 are chosen once for a network under the constraints that the total amount of each protein is conserved and the SN generated does have a stable inactive state.

The entries 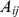 generate the index/indices for the reactant(s) 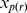 for the reaction 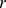. Each arc encoded by the interaction matrix is associated with a weight that represents the kinetic parameters with which production or degradation takes place. The dynamics 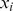 of the node 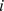 is governed by the equation:
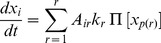
(1)

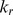
 denotes the kinetic parameter of the reaction number 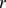. Note, that we chose 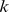 to be dimensionless in the sense that the time is scaled appropriately and the concentrations are normalized such that the numeric values of first- (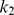, 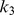 in Fig. 1) and second-order (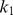 in Fig. 1) reactions approach a similar range.

The fitness of a SN was tested by calculating its response to 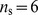 different signals. For every signal 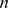, the dynamics of the pre-selected output node is tested whether it exceeds a threshold 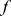. This threshold (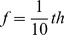 of the initial concentration level) is defined to be the relative fraction of the double phosphorylated protein to the total amount of the protein. If the output node crosses the threshold 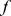 at any point during the dynamics the network gains a fitness contribution 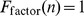. The normalized fitness 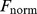 is calculated as the average fitness contribution for all signals which are weighted equally:
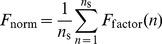
(2)Hence, the maximum fitness of a network will be 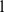 when it detects all signals or 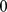 when there is no above-threshold response to any of the signals.

### 6.3 Algorithm

**Figure 10:**
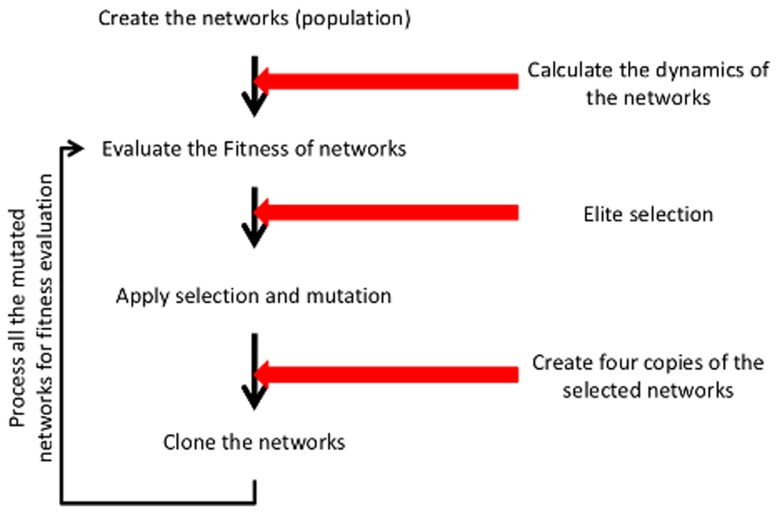
Scheme of the evolutionary algorithm. In order to generate genetic diversity, a set of 200 networks is created with diverse sets of kinetic parameters. In each iteration the dynamics of all networks is calculated for the complete set of input signals. Based on dynamics, the fitness is calculated. Based on the fitness scores, the successful network are selected (elite selection). For each selected network mutations are either applied to the kinetic parameters or the topology of the network by adding new proteins. Each selected network gives rise to an equal number of clones such that the population contains again 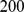 networks.

We have applied an evolutionary algorithm [28] to evolve the networks (Fig. 10). Before starting evolution, we create a set of diverse networks with the same randomly generated interaction matrix for three proteins with three states (un-, mono-, and dual-phosphorylated) and different randomly selected kinetic parameters (Eq. 1). The evolution of the networks is either controlled by mutation of the kinetic parameters or the addition of new nodes. In the latter case kinetic parameters are randomly selected once at the generation of the initial population and once for every single newly added protein.

The kinetic parameters are generated randomly initially in the range 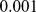 to 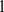. The total number of the networks is 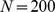. For each network, 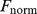 is computed. We perform elite selection of 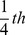 of the population. The successful networks are mutated by changing the kinetic parameters (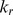) or adding new proteins as explained above. The subsequent generation is then populated by four copies of the successful networks keeping the number 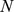 of networks identical in each generation. We evolve the population of networks for 200 generations. Systems of ordinary differential equations were formulated and solved with MATLAB 7.9.0.

## Supporting Information

Figure S1Kinetic parameter distribution of SNs before evolution. Randomly generated kinetic parameters between 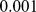 and 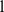 for all the simulations.(TIF)

Figure S2 Kinetic parameter distribution of SNs shortly after the fitness reaches maximum until the end of the simulation. Parameters are shown for the strong interaction regime (Sys III).
(TIF)

Figure S3List of possible reactions after the addition of a new node designated as 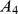 in the minimal model. 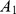, 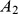, 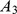, and 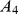 denote the inactive signaling proteins, their partially active (single phophorylated) forms are 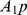, 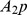, 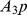, and 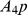, respectively and their fully active forms are 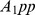, 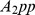, 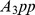, and 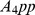, respectively.(TIF)
