# Supplementary figures and images for: Simulated Evolution of Signal Transduction Networks
Source: PLoS One. 2012 Dec 12;7(12):e50905. doi: 10.1371/journal.pone.0050905 (PMC3521023; doi:10.1371/journal.pone.0050905)

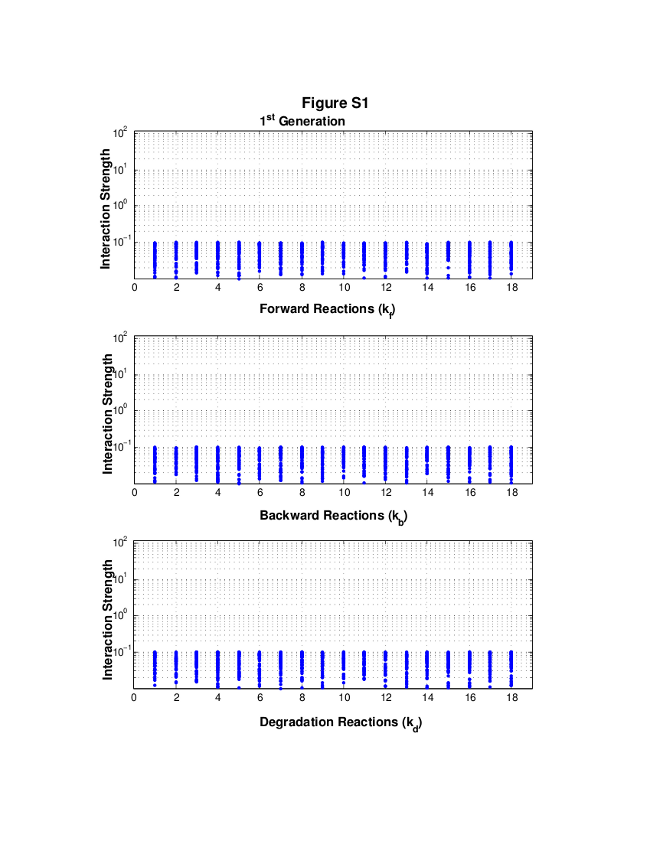

Supplement: Figure S1 — Kinetic parameter distribution of SNs before evolution. Randomly generated kinetic parameters between and for all the simulations. (TIF) [file pone.0050905.s001.tif]

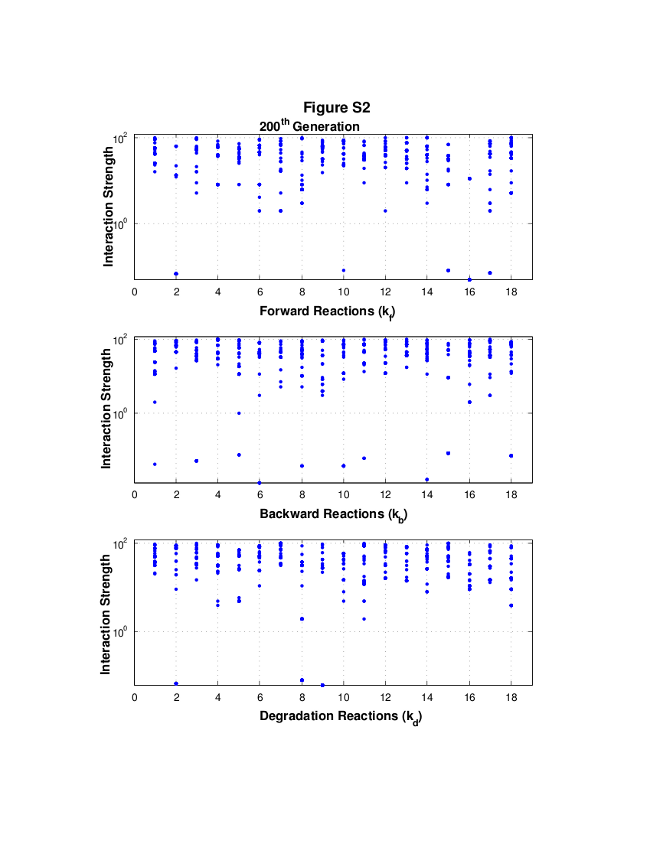

Supplement: Figure S2 — Kinetic parameter distribution of SNs shortly after the fitness reaches maximum until the end of the simulation. Parameters are shown for the strong interaction regime (Sys III). (TIF) [file pone.0050905.s002.tif]

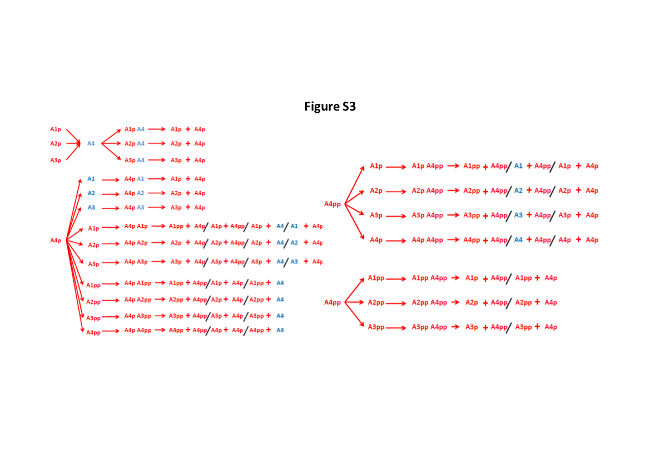

Supplement: Figure S3 — List of possible reactions after the addition of a new node designated as in the minimal model. , , , and denote the inactive signaling proteins, their partially active (single phophorylated) forms are , , , and , respectively and their fully active forms are , , , and , respectively. (TIF) [file pone.0050905.s003.tif]
